# Enzyme evolution: An annotated selection of World Wide Web sites relevant to the topics in microbial biotechnology

**DOI:** 10.1111/1751-7915.13329

**Published:** 2018-10-22

**Authors:** Lawrence P. Wackett

**Affiliations:** ^1^ Department of Biochemistry, Molecular Biology and Biophysics BioTechnology Institute University of Minnesota St. Paul MN 55108 USA

## Evolution of enzyme function

https://www.sciencedirect.com/science/article/pii/S0006349515004002

This review article takes the view of classifying enzymes and examining evolution within protein families.

## Enzyme evolution and engineering

https://www.ncbi.nlm.nih.gov/pubmed/29207314

This review article deals with natural enzyme evolution and application of the knowledge to engineer enzymes for biocatalytic applications.

## Directed evolution wins the Nobel Prize

https://www.chemistryworld.com/news/what-is-directed-evolution-and-why-did-it-win-the-chemistry-nobel-prize/3009584.article

This article from Chemistry World describes the Nobel Prize in Chemistry for 2018, with a focus on laboratory directed evolution methods developed by Frances Arnold and co‐workers.

## Chemistry and evolutionary aspects of enzymes

https://pubs.acs.org/doi/10.1021/acs.chemrev.8b00039

The “average” enzymes shows a *k*_*cat*_/K_M_ of ~10^5^ M^−1^ s^−1^ which is very much below diffusion limit. The issues underlying this phenomenon are discussed in this review.

## Evolutionary driver of enzyme thermal adapation

http://science.sciencemag.org/content/355/6322/289

In natural evolution over the span of earth life history, enzymes adapted to operate under lower temperature in most contemporary environments. The mechanisms of this adaption has relevance to understanding evolving enzymes for greater stability and temperature change.

## EASy method of laboratory enzyme evolution

https://www.genengnews.com/gen-news-highlights/easy-evolution-accelerates-enzyme-engineering/81255966

This news article describes a method of gene amplification and mutation to accelerate obtaining beneficial mutations.

## Biophysical model of enzyme evolution

https://www.biorxiv.org/content/early/2018/08/23/399154

This review takes a theoretical approach and concludes that the best fit for models suggest selection for stability and activity simultaneously.

## Enzyme evolution in the post‐genomic era

http://www.jbc.org/site/thematics/enzyme_evolution/

This page provides the overview for a special issue of the *Journal of Biological Chemistry* in which all of the articles deal with enzyme evolution.

## Directed evolution: Selection of the host organism

https://www.sciencedirect.com/science/article/pii/S2001037014600908

Most studies focus on the methods of DNA manipulation relevant to directed evolution. This review focuses on the choice of host organism to use for the procedures.

## *PLoS One* directed evolution papers

https://journals.plos.org/plosone/browse/directed_evolution

This page shows a collection of directed evolution papers in the journal.

## Directed evolution

https://sequencing.roche.com/en/technology-research/technology/directed-evolution.html

This Roche page highlights a primer on directed evolution and a link to relevant products.

## Directed evolution: Bioprocess lab

https://www.bsse.ethz.ch/bpl/research/directed-evolution.html

These pages provide good information and references pertaining to laboratory directed evolution.

## Frances H. Arnold group

http://fhalab.caltech.edu

This is the webpage for Nobel laureate Frances Arnold's laboratory with links to research, papers and laboratory personnel. Arnold's lab has been a leader in developing directed evolution methods.

## Directed evolution library creation

https://www.springer.com/us/book/9781493910526

This is a methods and procedures paper in a general methods book.

## Directed evolution and engineering of CRISP‐associated nucleases

https://dash.harvard.edu/handle/1/33840714

This page links to a doctoral thesis for the topic indicated.

## Biocatalysis in the pharmaceutical industry

https://www.ncbi.nlm.nih.gov/pmc/articles/PMC5430392/

Enzyme transformations related to pharmaceutical development is largely based on non‐natural reactions, and directed evolution is almost invariably required to generate enzymes with requisite specificity and rates.

